# Unilateral Duplication of the Foramen Spinosum With Unusual Bilateral Variation of Hypoglossal Canals in a Dry Human Skull: Their Clinical Implications

**DOI:** 10.7759/cureus.113508

**Published:** 2026-07-28

**Authors:** Tarun P Maheshwari, Keerthy Ajay Kumar

**Affiliations:** 1 Anatomy, All India Institute of Medical Sciences, Raebareli, Raebareli, IND

**Keywords:** duplication, foramen spinosum, hypoglossal canal, transcondylar approach, triplication

## Abstract

The foramen spinosum is one of the foramina of the skull that lies bilaterally close to the spine of the sphenoid in the middle cranial fossa. The recurrent meningeal branch of the mandibular nerve, middle meningeal artery, and vein usually passes through it. The hypoglossal canal, situated superior to the occipital condyles at the skull base, is a paired bony canal directed anterolaterally and serves as a conduit for the hypoglossal nerve, meningeal branch of the ascending pharyngeal artery, and emissary veins communicating with the basilar venous plexus. Duplication of the foramen spinosum along with duplication and triplication of the hypoglossal canal in the same skull is a rare osteological variant that may lead to variation in the course of contents. Recognition of such variations is of considerable clinical importance for neurosurgeons, skull base surgeons, and radiologists, as these anomalies may influence surgical planning and increase the risk of inadvertent neurovascular injury during procedures involving the middle and posterior cranial fossae. We report a case with duplication of the left foramen spinosum having a maximum diameter of 3.3 mm and 2.7 mm located just medial to the spine of the sphenoid. The same skull showed duplication of the left hypoglossal canal with a maximum extracranial diameter of 6.5 mm and triplication of the right hypoglossal canal with a maximum diameter of 7.6 mm located lateral to the occipital condyles. Coexistence of variation in the two types of foramina would certainly make it much clinically significant during skull base surgery to approach the middle meningeal artery and hypoglossal nerve in the middle and posterior cranial fossae, respectively. Surgeons should be cognizant about these variants while doing surgical procedures involving the middle meningeal artery and the transcondylar approach for the hypoglossal nerve to avoid further complications.

## Introduction

The foramen spinosum is a paired osseous opening located in the greater wing of the sphenoid bone, posterolateral to the foramen ovale in the middle cranial fossa. It typically transmits the middle meningeal artery and vein along with the recurrent meningeal branch of the mandibular nerve [1]. During cranial development, the foramen spinosum is formed by the progressive ossification of the posterior part of the greater wing of the sphenoid around the middle meningeal artery [2]. Duplication of the foramen spinosum has been attributed to an early extracranial division of the middle meningeal artery before its entry into the cranial cavity [3]. The hypoglossal canal, situated superior to the occipital condyles at the skull base, is a paired bony canal directed anterolaterally and serves as a conduit for the hypoglossal nerve, meningeal branch of the ascending pharyngeal artery, and emissary veins communicating with the basilar venous plexus [1]. Morphological variations of the hypoglossal canal are considered to have an embryological origin. These variations are believed to arise from the incomplete fusion of the occipital sclerotomes during skull base development, leading to the persistence of osseous septa that divide the hypoglossal canal into distinct compartments [4]. Anatomical variations involving these foramina, particularly in their number, morphology, and compartmentalization, may alter the course and distribution of associated neurovascular structures. Although duplication of the foramen spinosum and variations of the hypoglossal canal have been independently documented, the coexistence of a duplicated foramen spinosum with duplication and triplication of the hypoglossal canal within the same skull represents an exceedingly rare osteological finding. Recognition of such variations is of considerable clinical importance for neurosurgeons, skull base surgeons, and radiologists, as these anomalies may influence surgical planning and increase the risk of inadvertent neurovascular injury during procedures involving the middle and posterior cranial fossae.

## Case presentation

During routine osteology teaching sessions for undergraduate medical students at our institute, a rare anatomical variation involving the foramen spinosum and hypoglossal canal was identified in a dry adult male skull of unknown age and cause of death obtained from an institutional collection. The skull was intact, well preserved, and devoid of any gross pathological changes, traumatic defects, or congenital deformities that could interfere with the assessment of skull base foramina. The measurements of the openings were recorded using a carbon fiber digital vernier caliper of ±0.1 mm accuracy.

**Figure 1:**
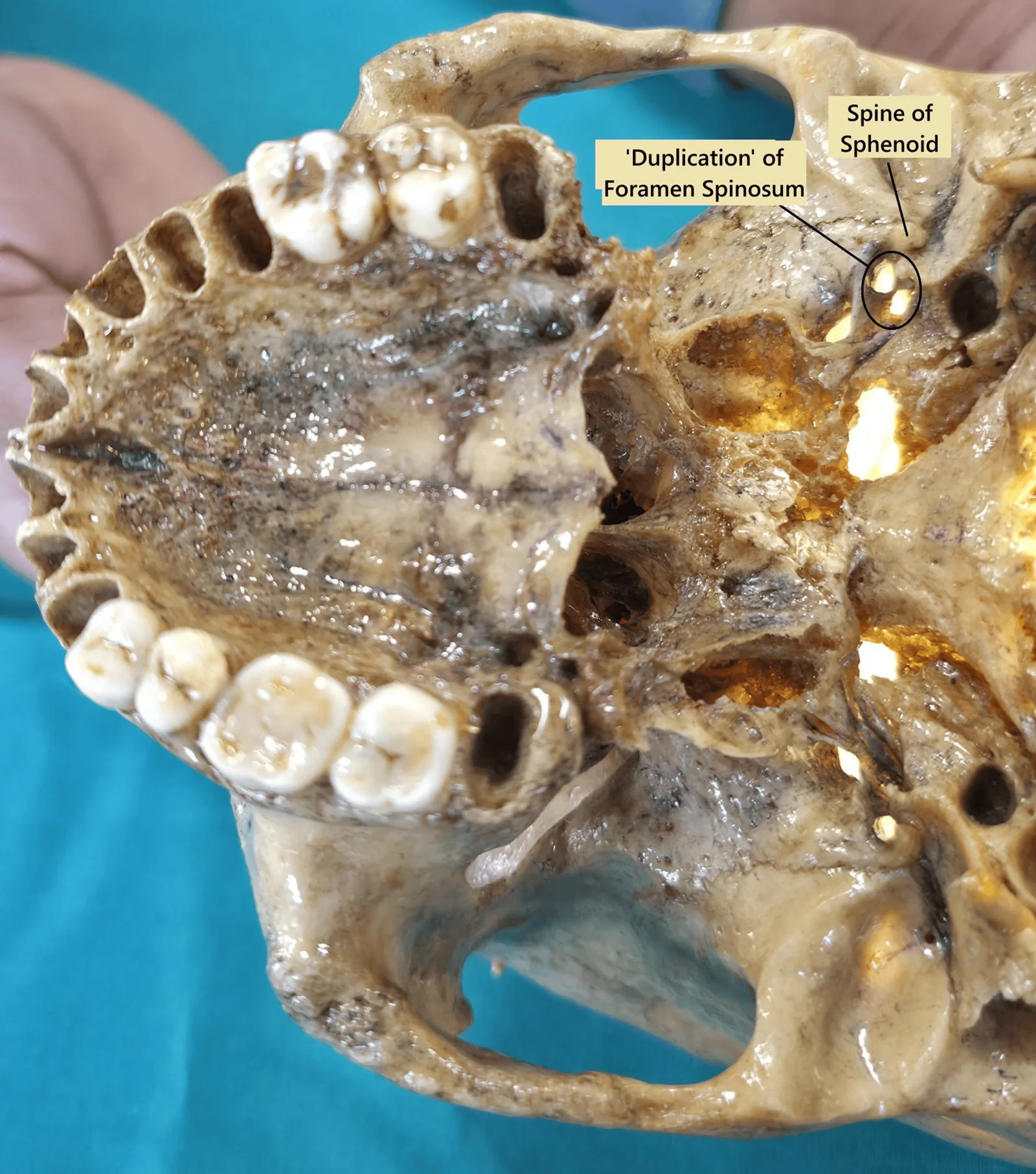
Base of a dry human skull showing duplication of the foramen spinosum

Morphological examination revealed duplication of the left foramen spinosum with two distinct foramina (Figure 1), measuring 3.3 mm and 2.7 mm in maximum diameter, respectively. The duplicated foramen spinosum was oval in shape and located posterolateral to the foramen ovale and medial to the spine of the sphenoid. The contralateral side demonstrated a single normally positioned foramen spinosum that was round in shape. The surrounding osseous structures appeared normal.

**Figure 2:**
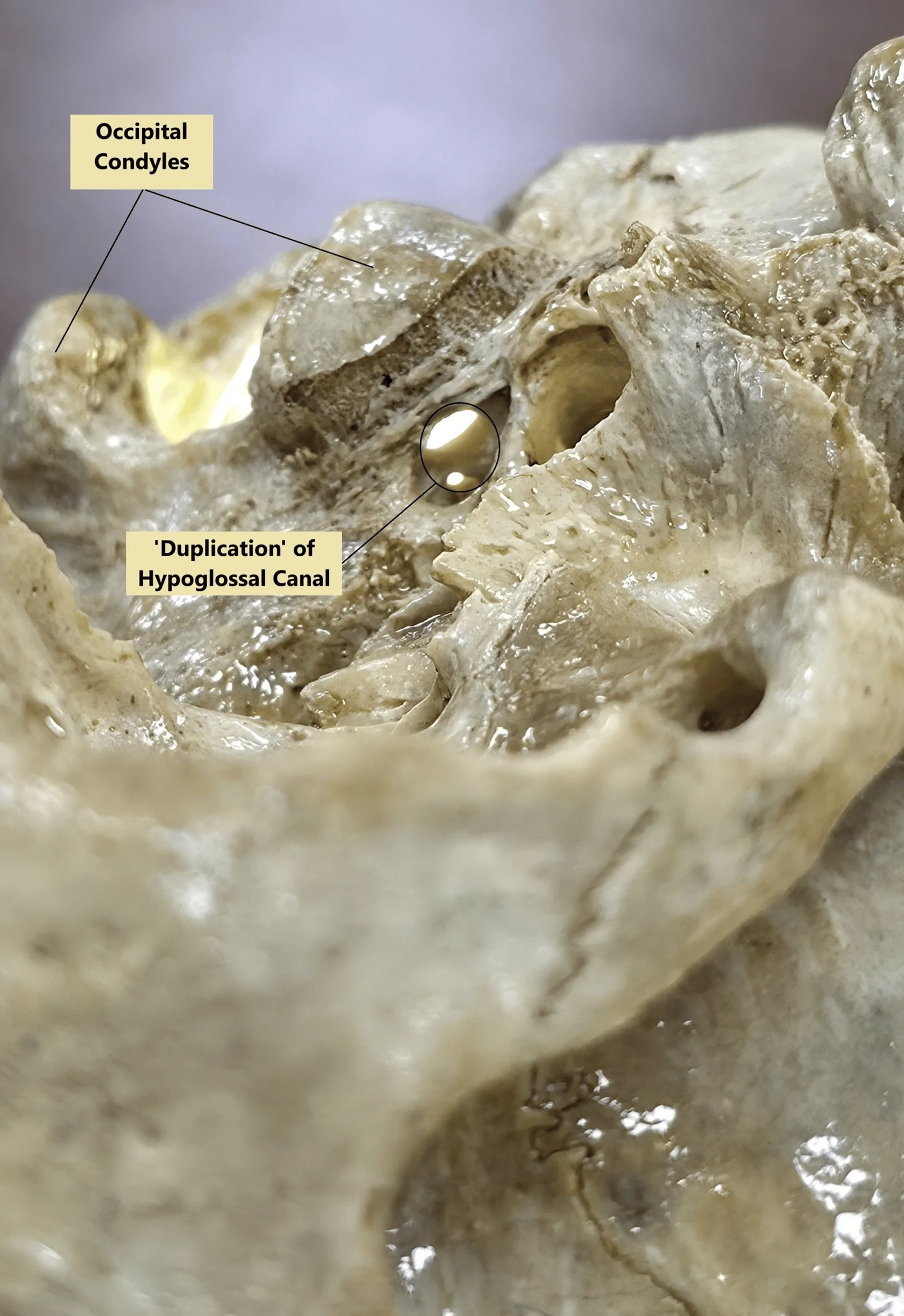
Base of a dry human skull showing duplication of the left hypoglossal canal

**Figure 3:**
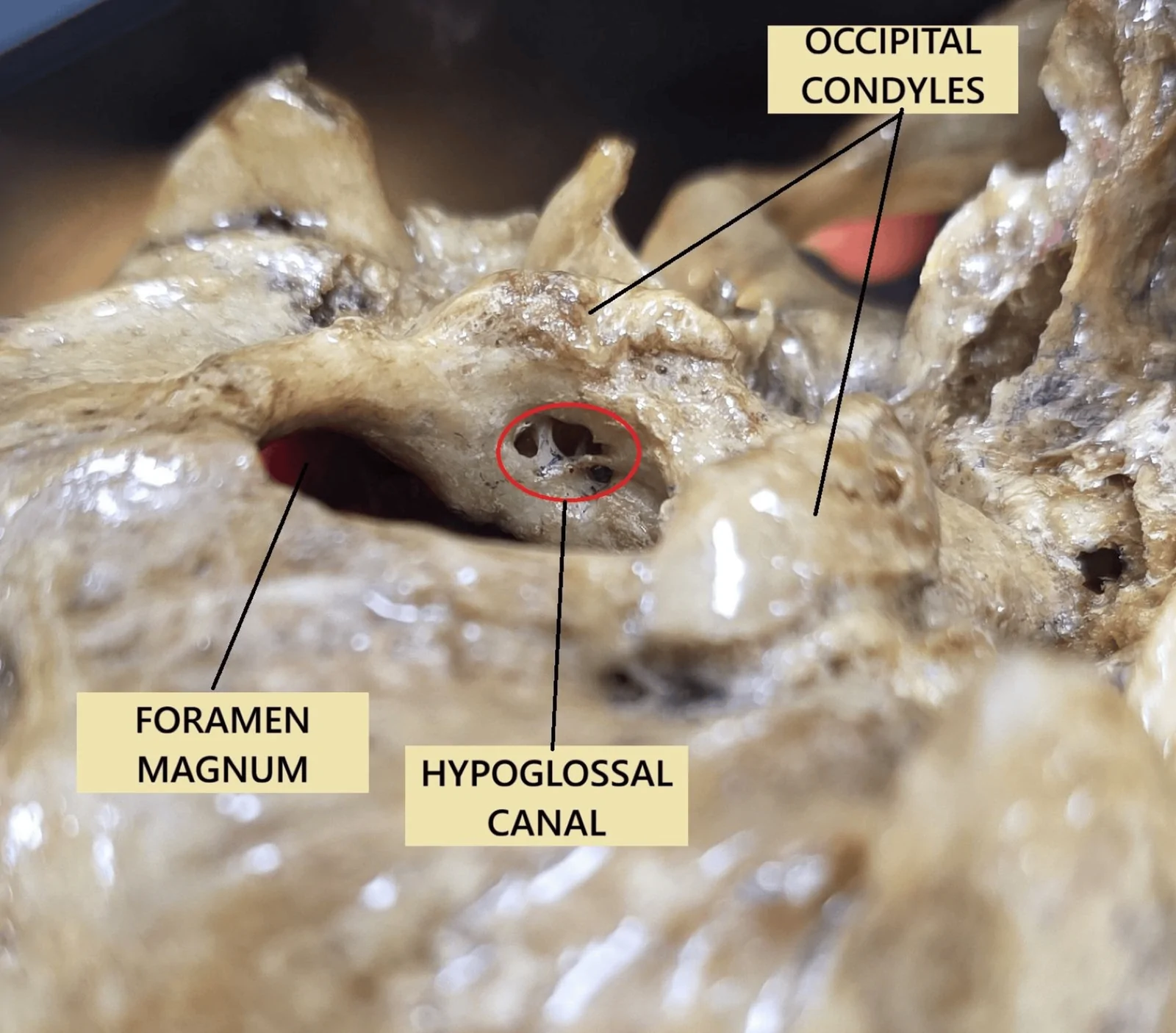
Base of a dry human skull showing triplication of the right hypoglossal canal

Furthermore, the same skull demonstrated asymmetrical variations of the hypoglossal canal, characterized by duplication on the left side (Figure 2) and triplication on the right side (Figure 3). The osseous septum appeared complete and robust, indicating complete partitioning of the canal rather than partial bridging. The duplicated left hypoglossal canal exhibited a maximum extracranial diameter of 6.5 mm, whereas the triplicated right hypoglossal canal measured 7.6 mm in maximum diameter. These canals were located lateral to the occipital condyles. No associated abnormalities of the occipital condyle, jugular foramen, or foramen magnum were identified.

## Discussion

The foramen spinosum exhibits considerable anatomical variability in its morphology, number, and dimensions, reflecting the complex developmental processes involved in skull base ossification. Although morphometric studies on the foramen spinosum have extensively documented its size, shape, and topographical relationships [5,6], reports describing duplication of the foramen spinosum remain relatively scarce. Duplication of the foramen spinosum is generally attributed to developmental alterations in the course or branching pattern of the middle meningeal artery during ossification, resulting in the formation of accessory osseous canals.

Similarly, variations of the hypoglossal canal are predominantly associated with the formation of complete or incomplete bony septa during the endochondral ossification of the occipital bone, leading to duplication, triplication, or compartmentalization of the canal [7,8]. Such developmental modifications may alter the arrangement of neurovascular structures traversing the canal, including the hypoglossal nerve, meningeal branch of the ascending pharyngeal artery, and emissary venous communications. In a notable osteological study, Raghunath et al. documented a rare case of bilateral internal triplication of the hypoglossal canal in a dry human skull, emphasizing the rarity of such variants [9].

From a clinical perspective, anatomical variations involving the hypoglossal canal possess substantial surgical significance, particularly in skull base interventions such as condylectomy and transcondylar approaches to the posterior cranial fossa [10-12]. Precise knowledge of the morphology and topographical disposition of hypoglossal canal orifices and variations of the foramen spinosum may have implications in neurosurgical and radiological procedures to avoid inadvertent injury during surgical exposure. Such a documented case of duplication of the foramen spinosum along with triplication and duplication of the hypoglossal canal should be considered significant.

## Conclusions

The coexistence of a duplicated left foramen spinosum with unilateral duplication and contralateral triplication of the hypoglossal canal in the same skull, as observed in the present case, represents an exceedingly rare osteological variation. To the best of our knowledge, such a combination of cranial foraminal variations has been infrequently reported in the literature. Therefore, awareness and documentation of these unusual anatomical configurations are important for anatomists, radiologists, and skull base surgeons to facilitate the accurate interpretation of radiological findings and optimize surgical planning.
